# Re-visiting the nature and relationships between neurological signs and neurocognitive functions in first-episode schizophrenia: An invariance model across time

**DOI:** 10.1038/srep11850

**Published:** 2015-07-02

**Authors:** Raymond C. K. Chan, Shan Dai, Simon S. Y. Lui, Karen K. Y. Ho, Karen S. Y. Hung, Ya Wang, Fu-lei Geng, Zhi Li, Eric F. C. Cheung

**Affiliations:** 1 Neuropsychology and Applied Cognitive Neuroscience Laboratory, Key Laboratory of Mental Health, Institute of Psychology, Chinese Academy of Sciences, Beijing, China; 2 Department of Statistics and Finance, University of Science and Technology of China, Hefei, China; 3 University of Chinese Academy of Sciences, Beijing, China; 4 Castle Peak Hospital, Hong Kong Special Administrative Region, China

## Abstract

The present study examined different types of neurological signs in patients with first-episode schizophrenia and their relationships with neurocognitive functions. Both cross-sectional and longitudinal designs were adopted with the use of the abridged Cambridge Neurological Inventory which comprises items capturing motor coordination, sensory integration and disinhibition. A total of 157 patients with first-episode schizophrenia were assessed at baseline and 101 of them were re-assessed at six-month interval. A structural equation model (SEM) with invariance model across time was used for data analysis. The model fitted well with the data at baseline assessment, X^2(21) = 21.78, p = 0.413, NFI = 0.95, NNFI = 1.00, CFI = 1.00, IFI = 1.00, RMSEA = 0.015. Subsequent SEM analysis with invariance model at six-month interval also demonstrated the same stable pattern across time and showed strong measurement invariance and structure invariance across time. Our findings suggest that neurological signs capture more or less the same construct captured by conventional neurocognitive tests in patients with schizophrenia. The measurement and structure of these relationships appear to be stable over time.

Neurological soft signs (NSS) have long been observed in schizophrenia spectrum disorders and have been considered possible “target features” or endophenotypes^1^,^2^. Substantial evidence has shown that NSS capturing motor coordination, sensory integration, complex motor sequencing and disinhibition are significantly and consistently increased in patients with schizophrenia compared to healthy controls across different stages of the illness^3^,^4^,^5^,^6^,^7^,^8^,^9^,^10^. Recent imaging findings also provide strong evidence that NSS are associated with specific brain structural and functional connectivity deficits corresponding to clinical manifestations in patients with schizophrenia^11^,^12^.

The brevity of NSS assessment (less than 10 minutes) makes it feasible for clinicians and researchers to screen for neurocognitive dysfunction in patients with schizophrenia. Chan and Gottesman^13^,^14^,^15^ have argued that both NSS and conventional neurocognitive tests capture the same underlying brain functions that can be linked both at the microscopic (genetic components) and macroscopic (clinical syndromes) levels in schizophrenia spectrum disorders. These arguments have been substantiated by empirical findings studying the relationship between NSS and neurocognitive functions (e.g.^9^,^16^,^17^,^18^). Adopting a rigorous structural equation modeling (SEM) approach, Chan *et al.*^18^ have demonstrated that both the measurement and structural models fitted well with the data in both patients with established schizophrenia and healthy controls. More importantly, there were modest to strong associations between NSS, executive functions, and memory in both schizophrenia patients and healthy controls independently. These findings suggest that these apparently distinct constructs are actually capturing similar higher cortical functions in patients with schizophrenia. However, these findings were limited to patients with a relatively long duration of illness and prolonged medication exposure. It is possible that these findings were confounded by illness chronicity and medication effects.

The purpose of the present study was to examine the relationship between NSS and conventional neurocognitive functions in patients with first-episode schizophrenia. One significant distinction of the present study from previous ones is that we adopted the invariance model in SEM to examine the temporal stability of the latent variables identified from NSS evaluation in a large sample of first-onset schizophrenia patients. This approach allowed us to remove the confounding effects of illness duration and medication exposure. To the best of our knowledge, this is the first study examining the temporal stability of NSS in a large sample of patients with first-episode schizophrenia. We hypothesized that NSS capture the same level of cortical function measured by conventional neurocognitive tests, and that these structure and measure models derived from the SEM are stable over time.

## Results

One hundred and fifty-seven (75 men) patients with first-episode schizophrenia were recruited. Of these, 101 (50 men) were followed up six months later. The mean age and years of education were 24.39 years (SD = 6.12), 11.61 years (SD = 2.15) at baseline and 24.51 years (SD = 6.23), 11.60 years (SD = 2.11) at the six-month time point. The mean duration of untreated psychosis was 3.63 months (SD = 4.99). The groups at the two time points did not differ in age, education, and gender proportion. Table 1 summarizes the prevalence rate of individual items of NSS in patients and healthy controls. A small proportion of NSS items were already present in the first presentation of schizophrenia, e.g., finger agnosia, fist-edge-palm and left-right disorientation. By paired T-test analysis for NSS and PANSS data across the two time points, we found significant reduction in motor coordination, sensory integration and total NSS scores (p = 0.05), but there was no significant reduction in disinhibition signs. Similarly, there were also significant reduction in positive symptoms and general psychopathological symptoms on the PANSS between the two time points (p = 0.05), but there was no significant reduction in negative symptoms. In contrast, there was significant improvement in almost all of the neurocognitive functions (p = 0.05), except verbal fluency and visual reproduction-delayed recall. These results suggest that these patients were at least in partial remission at the time of re-assessment.

### SEM at the first observation time point

**Figure 1:**
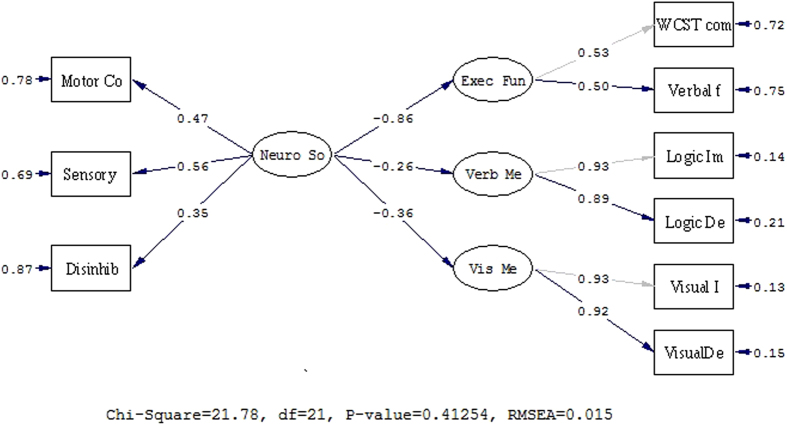
The structure model at baseline. X^2(21) = 21.78, p = 0.413, NFI = 0.95, NNFI = 1.00, CFI = 1.00, IFI = 1.00, RMSEA = 0.015.

The results showed that the four-factor measurement model fitted the data well. Almost all of the loadings of the observed variables of corresponding latent variables were above 0.4 and statistically significant (p < 0.01, see Fig. 1), except the loading of NSS on disinhibition. Thus, almost all of the latent variables appeared to have been adequately measured by their respective observed variables. Furthermore, correlations between the independent latent variable (NSS) and dependent latent variables (i.e. attention/executive function, logical memory and visual memory) were all statistically significant (p < 0.01, see Fig. 1 and Table 2). Concerning the structural model, the model fitted well with the data at baseline (X^2(21) = 21.78, p = 0.413, NFI = 0.95, NNFI = 1.00, CFI = 1.00, IFI = 1.00, RMSEA = 0.015). Moreover, the modification indices given by LISREL also suggested that the original model was a good fit (Fig. 1). All these results suggest that NSS had important negative influence on executive attention, verbal memory, and visual memory. Higher level of NSS was associated with more severe impairment of executive attention and memory functions. On the basis of the above, we carried out SEM analysis with invariance model on the data collected six months later in 101 patients with first-episode schizophrenia.

### Exploring the measurement equivalence of SEM over time

Applying SEM analysis with invariance model over time on the data which included assessment results of the same 101 patients at the six-month time point, we obtained the fit indices for the SEM with invariance model over time displayed in Table 3.

To determine significant differences between the tested models we adopted a model in which a ΔNNFI ≥ −0.010, ΔCFI ≥ −0.010, supplemented by ΔRMSEA ≥ 0.015 would be indicative of non-invariance^19^,^20^,^21^. The initial model that assessed configural invariance showed that the main fit index were as follows: X2(104) = 121.05, NNFI = 0.981, CFI = 0.987, RMSEA = 0.040. The result showed a good fit, which suggested that the SEM model had the same pattern over time. These results provided a basis for further tests, and showed that the constructs could be conceptualized in the same way across time^22^.

The second step was the test of metric invariance across time. The model also yielded an acceptable fit: according to the evaluation criterion stated earlier, the result of ΔNNFI = −0.005, ΔCFI = −0.005, ΔRMSEA = 0.005, indicated that the SEM model had the same factor loadings across time, which reflected the same metric of the measurement structure across time.

Similar to the second step test, the model fit index yielded an acceptable result in the test of scalar invariance. The scalar invariance combined with the metric invariance indicated that the metric and reference substance across time were stable, suggesting that the constructs measured by the test items were unbiased^23^.

**Figure 2:**
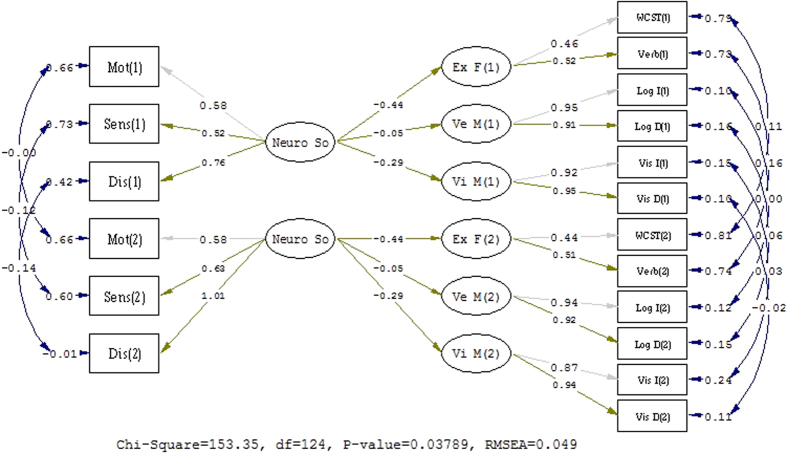
The structure model of test for Factor variance and covariance invariance of measurement invariance. X^2(124) = 153.35, p = 0.038, NNFI = 0.97, CFI = 0.98, IFI = 0.98, RMSEA = 0.

**Figure 3:**
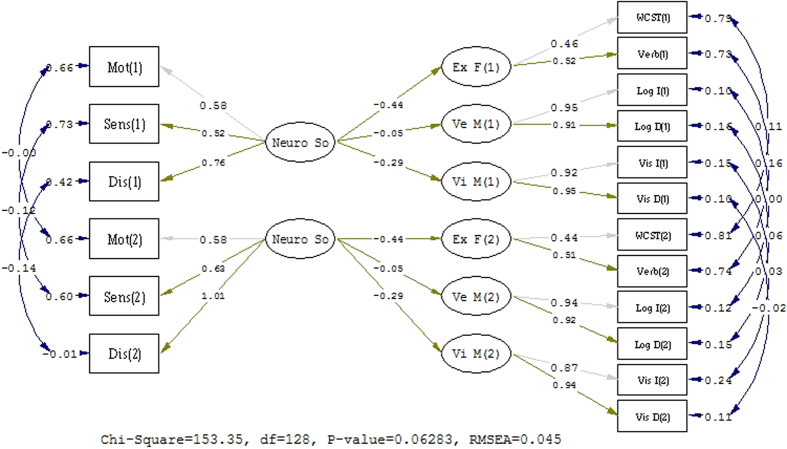
The structure model of test for Latent mean invariance of measurement invariance. X^2(128) = 153.35, p = 0.063, NNFI = 0.98, CFI = 0.98, IFI = 0.98, RMSEA = 0.045.

On the basis of the third step test, we constrained the residual variances to be the same across time, and the model fit of ΔNNFI = −0.013, ΔCFI = −0.012, ΔRMSEA = 0.013, was not unacceptable^19^,^21^. Concerning the tests of invariant factor variances and invariant factor covariances, the model fit was good (see Fig. 2) according to our evaluation criterion. The invariant factor variances and invariant factor covariances showed stable factor structure, which implied similar heterogeneity of constructs over time and similar relationships between constructs over time. Figure 3 further elucidated the latent mean invariance with a good fit of index and indicated that the latent mean of the constructs measured in the SEM model was relatively stable across a six-month interval.

## Discussion

Our results are consistent with our previous findings on the relationship between NSS and conventional neurocognitive functions in patients with chronic schizophrenia^18^. In the present sample with first-episode schizophrenia patients, we found a good fit of the measurement and structural models of the SEM showing that NSS capture approximately the same constructs captured by conventional neurocognitive tests. The robust relationship is supported by the moderate to high regression coefficients between NSS, executive attention and memory functions. A higher level of NSS was associated with more severe impairment measured by conventional neurocognitive functions. These findings are also consistent with those found in healthy volunteers and healthy ageing people^15^,^18^.

More importantly, our results extended the examination of the relationship between NSS and conventional neurocognitive functions across two time points. This approach allows examination of the temporal stability of these relationships over six months after the first onset of schizophrenia. Our findings showed that significant and stable relationships exist between NSS and conventional neurocognitive functions in patients with first-episode schizophrenia. However, it should be noted that there were significant decrease in NSS scores in the course of the illness in the present study, mainly in motor coordination and sensory integration signs. This may be due to several reasons. First, it may be due to practice effect across the two time points, which is especially important for the motor coordination items involving complex motor sequencing. Moreover, the decrease in NSS scores may reflect a “regression to the mean” phenomenon in the measurement. Verification with a healthy control group is needed. Finally, the improvement in NSS scores might have been inflated by the large sample size in the present study. Most of the previous longitudinal studies of NSS in first-episode schizophrenia were limited by small sample size^24^,^25^. Indeed the effect size and mean score of the motor coordination and sensory integration signs in the present study were small in magnitude (cohen’s d <0.2) and there was only a modest change in absolute scores between the two time points. Nevertheless, to the best of our knowledge, this is the first study that uses a rigorous approach like SEM to examine the temporal stability of the relationships between NSS and conventional neurocognitive functions in first-episode schizophrenia.

Our findings have two important implications. First, the significant and robust association between NSS and neurocognitive functions suggests a common neural substrate between these two constructs. Empirical findings from structural and functional imaging studies in healthy and schizophrenia samples also support this hypothesis^12^. Conventionally, evaluation of cognitive functions using either specific individual tests or a battery of tests is time-consuming and requires considerable training. The evaluation of NSS, on the other hand, only takes 10 minutes and requires relatively brief training to achieve high inter-rater reliability^18^,^26^, making it a much more convenient bedside screening assessment for neuropsychiatric disorders.

Secondly, NSS have long been considered a possible endophenotype for schizophrenia. A recent study by the Consortium on the Genetics of Schizophrenia (COGS) has shown that the heritability estimate of schizophrenia is equivalent to the mean heritability estimates of the 12 endophenotypes of 30% with seven of the COGS endophenotypes exhibiting higher heritability estimates than the diagnosis of schizophrenia in nuclear families^27^. However, the COGS has not included NSS in their assessment battery but only included one related item, namely sensory motor dexterity. This item was found to be associated with NRG1 and GRM2^28^ and was the only item reaching critical threshold in a genome-wide linkage analysis study^29^. Genetic studies from schizophrenia also suggest that the catechol-O-methytransferase (COMT), the GRM3 genetic variations, and seven nicotinic cholinergic receptors^30^,^31^ may be associated with NSS. The utility of endophenotypes lies in the presumably “cleaner” and measurable signals they produce as a result of being more closely related to the underlying neurobiological processes, compared to the conglomeration of traits and symptoms embedded in the diagnosis^27^.

The present study has several limitations. Although the baseline model produced a good model fit, it is possible that other constructs could be established to depict the relationship between NSS and neurocognitive functions. Future studies should examine more effective SEM model that reflects the relationship between NSS and neurocognitive functions more precisely. Secondly, the re-assessment sample consisted of only 101 of the total sample, which might have led to volatility of the results. Thirdly, the theory of SEM with invariance model across time is incomplete, which adds to the difficulty for us to establish more accurate model to test the measurement invariance. The additional use of partial invariance theory^32^ to analyze and interpret the SEM data may provide a more precise method to improve our findings^22^. Finally, the present study did not recruit a healthy control group to examine the natural variation of NSS in healthy individuals.

Notwithstanding these limitations, the present study provides one of the most rigorous findings on the stability of the structural and measurement models of NSS and conventional neurocognitive functions in a relatively large sample of patients with first-episode schizophrenia. These findings extend and replicate our previous findings on patients with established schizophrenia. The brevity and ease of administration of NSS assessment compared with conventional neurocognitive tests makes it a useful alternative for neurocognitive function screening in the clinical settings.

## Methods

### Participants

Patients with first-episode schizophrenia were recruited from the joint research-based first-episode schizophrenia programme between Castle Peak Hospital of Hong Kong and the Key Laboratory of Mental Health, Institute of Psychology, Chinese Academy of Sciences in Beijing^33^. All patients fulfilled the diagnostic criteria of schizophrenia based on the DSM-IV^34^, ascertained using the Structured Clinical Interview for DSM-IV and medical record reviews. We adopted the best-estimate approach in ascertaining the diagnosis using structured interview and case record review. We confirmed the diagnosis at the follow-up time point (six months later) with the DSM-IV^34^. Exclusion criteria included physical illness involving the central nervous system, substance and/or alcohol abuse, and clinical evidence of mental retardation. Clinical symptoms were assessed using the Positive and Negative Syndrome Scale (PANSS^35^). The study was approved by the Ethics Committees of the New Territories West Cluster of the Hospital Authority of Hong Kong and the Institute of Psychology, the Chinese Academy of Sciences in Beijing. The methods were carried out in accordance with the approved guidelines. Informed consent was obtained from all the participants prior to testing.

### NSS Assessment

NSS assessment was performed by experienced psychiatrists (SSYL, KKYH, KSYH) using the abridged version of the Cambridge Neurological Inventory (CNI)^18^. This abridged version offers instructions for eliciting and rating a comprehensive range of NSS, namely motor coordination, sensory integration, and disinhibition. Item scores were scored as “absent” (covering normal or equivocal scores) or “present” (covering abnormal or grossly abnormal scores). Each item score was summed up to a subscale score for motor coordination, sensory integration and disinhibition and a total score of NSS. A higher score indicated a higher level of NSS. Inter-rater reliability was calculated for each of the subscales based on investigators’ ratings on 10 participants not involved in this study using intraclass correlation: motor coordination (0.91), sensory integration (0.85) and disinhibition (0.9).

### Neurocognitive assessment

Executive function was assessed by the modified version of the Wisconsin Card Sorting Test (WCST)^36^ and the Verbal Fluency Test^37^. The main difference between the modified version and the original version of the WCST^38^ is that the participants were informed about the change of rule of card sorting criteria in the modified version. Verbal memory and visual memory were measured by the Logical Memory Subscale and the Visual Reproduction Subscale of the Chinese version of the Wechsler Memory Scale-Revised version^39^. These cognitive assessments covered the conventional domains of attention, memory and executive functions. These assessments were exactly the same as the previous study we conducted in a sample of patients with chronic schizophrenia^18^, thereby allowing comparison of findings between the two studies.

### Procedure

Trained psychiatrists carried out the NSS assessment. Intellectual functioning was assessed by the short form of the Chinese version of the Wechsler Adult Intelligence Scale-Revised (WAIS-R)^40^. This method of prorating has previously been used in estimating intellectual functioning in schizophrenia^41^,^42^.

### Data analysis

Structural equation model (SEM) and tests of measurement invariance with invariance model was conducted with LISREL 8.80 for Windows.

### SEM

SEM is widely used to test and estimate causal relationship in social sciences in order to isolate observational error from measurement of latent variables. It mainly consists of the measurement model and the structural model. The structural model shows potential causal dependencies between endogenous and exogenous variables, and the measurement model shows the relationships between latent variables and their indicators. Using a combination of inputted correlation matrix data and causal assumptions^43^, we obtained a fitted model of which the estimated covariance matrices best approximate the actual covariance matrices and thus best represent the relationships between variables, defined by fit indices.

In the present study, the measurement model was developed based on the previous four-factor model we found in patients with chronic schizophrenia^18^. It consists of executive function, verbal memory, visual memory and NSS. The structural model tests the relationships between NSS and the three conventional neurocognitive functions. The validity of the model was tested with chi-square test and five fit indices, namely the Normed Fit Index (NFI), the Non-Normed Fit Index (NNFI), the Comparative Fit Index (CFI), the Incremental Fit Index (IFI), and the Root Mean Square Error of Approximation (RMSEA). A value of .90 or above for the first four fit indices, and a value of .08 or less for the RMSEA indicate that the model adequately fits the data^44^,^45^.

### Measurement invariance of SEM

The SEM with invariance model across time adopted by us provides a rigorous method to test measurement invariance and to explore the stability of the relationship between NSS and neurocognitive functions across two time points. Measurement invariance works by running a set of increasingly constrained Structural Equation Models, and then testing whether differences between these models are significant. It consists of two types of invariance^32^, namely measure invariance: consisting of configural invariance, metric invariance, scalar invariance and error variance invariance; and structure invariance: consisting of factor variances invariance, factor covariance invariance and latent mean invariance. The specific invariance tests and their meanings and methods^19^,^23^ are shown in Table 3.

Different from measurement invariance in the usual multi-group confirmatory factor analysis (CFA), the tested longitudinal measurement invariance in the SEM with invariance model across time sets the same measured items to be correlated across time^46^.

## Additional Information

**How to cite this article**: Chan, R.C.K. *et al.* Re-visiting the nature and relationships between neurological signs and neurocognitive functions in first-episode schizophrenia: An invariance model across time. *Sci. Rep.* **5**, 11850; doi: 10.1038/srep11850 (2015).

## Figures and Tables

**Table 1:** Description of the samples at baseline and matched follow-up at 6-month interval.

	Patients at baseline (N = 157) (Mean, SD)	Patients at baseline (N = 101) (Mean, SD)	Patients at 6-month interval (N = 101) (Mean, SD)	T-tests
Age (years)	24.39,6.12	24.51,6.23	24.51,6.23	–
Education (years)	11.61,2.15	11.60,2.11	11.60,2.11	–
Gender (F:M)	82:75	51:50	51:50	
Duration of illness (months)	3.63,4.99	3.55,5.26	–	–
Medication(Chlorpromazine equivalence) (mg/day)	1.49,2.14	1.65,2.36		–
PANSS		–		–
Positive symptoms	11.40,4.74	10.98,4.37	8.38,2.92	5.29*
Negative symptoms	12.42,5.78	12.33,6.10	11.53,6.12	1.52
General psychopathology	22.96,6.45	22.80,6.69	19.73,4.85	4.10*
NSS scores	6.10,3.11	5.83,3.12	4.72,2.98	4.00*
Motor coordination	2.80,1.88	2.68,1.90	1.92,1.75	4.11*
Sensory integration	2.11,1.46	2.00,1.44	1.46,1.31	3.62*
Disinhibition	1.18,1.06	1.15,1.09	1.35,1.14	–1.67
Neurocognitive Functions		–	–	–
WCST categories	5.08,1.53	4.96,1.62	5.65,1.05	–4.36*
Verbal fluency	17.22,4.76	17.33,4.80	17.74,5.45	–0.80
Logical memory-immediate recall	7.88,3.57	7.83,3.41	9.17,3.80	–4.77*
Logical memory- delayed recall	5.99,3.44	5.99,3.61	7.51,3.83	–5.53*
Visual reproduction- immediate recall	20.38,3.12	20.61,3.08	20.82,3.09	–0.71*
Visual reproduction- delayed recall	19.82,3.45	20.08,3.30	20.01,4.03	0.20

Note:*p < 0.05

**Table 2:** Tests for measurement invariance across time.

Model	df	Δdf	Δ 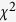	P	NNFI	ΔNNFI	CFI	ΔCFI	RMSEA	ΔRMSEA
M1 Configural invariance	104	—	—	0.121	0.981	—	0.987	—	0.040	—
M2 Metric invariance	112	8	14.101	0.067	0.976	−0.005	0.983	−0.005	0.045	0.005
M3 Scalar invariance	117	5	0	0.120	0.982	0.006	0.986	0.004	0.039	−0.006
M4 Error variance invariance	126	9	24.873	0.022	0.969	−0.013	0.974	−0.012	0.052	0.013
M5 Invariant factor variances	121	4	11.219	0.058	0.976	−0.006	0.981	−0.006	0.046	0.006
M6 Invariant factor covariance	124	3	6.977	0.038	0.973	−0.003	0.978	−0.003	0.049	0.003
M7 Latent mean invariances	128	4	0	0.063	0.977	0.005	0.981	0.003	0.045	−0.004

Note: df – Degrees of freedom; Δdf –change in degrees of freedom,CFI - Comparative Fit Index; ΔCFI- change in Comparative Fit Index; NFI- Nonnormed Fit Index; ΔNNFI - change in Nonnormed Fit Index; RMSEA = Root Mean Square Error of Approximation; ΔRMSEA = change in Root Mean Square Error of Approximation.

Nested models were tested to determine whether the structure of neurological signs and conventional neurocognitive tests were operating equivalently across time.

**Table 3:** Constraints and steps of measurement invariance.

Tests	Constraints	Meaning	Interpretation
Configural invariance	No constraints	Same pattern	Same model structure across time
Metric invariance	Λ^1^ = Λ^2^=…	Equally constrained matrices of factor loadings	Same metric across time
Scalar invariance	*τ*^1^ = *τ*^2^=…	Equally constrained vector with item intercepts	Same systematic response bias across time
Invariance of error variances	Θ^1^ = Θ^2^=…	Equally constrained matrix with residuals variances	Same internal consistency across time
Invariance of factor variances	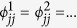	Equivalence of construct variance	Same heterogeneity of constructs across time
Invariance of factor covariance	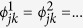	Equivalence of construct covariance	Same relationships among constructs across time
Invariance of latent means	*κ*^1^ = *κ*^2^=…	Equivalence of latent means	Same mean level of each constructs across time
